# Inflammation and nutrition-derived indicators for predicting amputation risk in patients with type 2 diabetic foot ulcers

**DOI:** 10.3389/fnut.2026.1875176

**Published:** 2026-06-30

**Authors:** Junyu He, Jingxia Sun, Jinhua Wu, Jingming Yu, Lili Zhu, Wensheng Lu

**Affiliations:** 1 Department of Endocrinology and Metabolism, Guangxi Academy of Medical Sciences and the People’s Hospital of Guangxi Zhuang Autonomous Region, Nanning, Guangxi, China; 2 Department of Gastroenterology, Guangxi Academy of Medical Sciences and the People’s Hospital of Guangxi Zhuang Autonomous Region, Nanning, Guangxi, China; 3 Department of Enterprise Management, China Tobacco Guangxi Industrial Company Limited, Nanning, Guangxi, China

**Keywords:** amputation, diabetic foot ulcer, indicators, inflammation, machine learning, nutrition

## Abstract

**Background:**

Inflammation and malnutrition affect the prognosis of diabetic foot ulcers (DFU). This study systematically evaluated the impact and predictive value of inflammation and nutrition-derived indicators for amputation in DFU using machine learning models.

**Methods:**

The study enrolled 1,052 patients with type 2 diabetic foot ulcers. During hospitalization, 739 patients received conservative treatment and 313 patients underwent amputation, including 259 patients who received minor amputation and 54 patients who received major amputation. Multivariate logistic regression analysis was used to investigate the correlation between the inflammation and nutrition-derived indicators and amputation in DFU. Restricted cubic splines (RCS) and receiver operating characteristic (ROC) curves were utilized to evaluate the relationship and predictive value of derived indicators with the risk of amputation. Five machine learning algorithms (logistic regression, random forest, support vector machine (SVM), extreme gradient boosting (XGBoost), and light gradient boosting machine (LightGBM)) were used to construct predictive models for amputation in DFU. The SHapley Additive exPlanations (SHAP) method was applied to analyze the contribution of each feature in the model to the predictive outcomes.

**Results:**

Multivariate logistic regression and RCS analysis demonstrated that systemic inflammatory response index (SIRI), neutrophil-lymphocyte ratio (NLR), platelet-lymphocyte ratio (PLR), neutrophil-high-density lipoprotein (HDL) ratio (NHR), monocyte-HDL ratio (MHR), platelet-HDL ratio (PHR), and neutrophil-albumin ratio (NAR) were positively correlated with amputation in DFU. The platelet–neutrophil ratio (PNR), lymphocyte-white blood cell ratio (LWR), and the hemoglobin, albumin, lymphocyte, and platelet (HALP) score were negatively associated with amputation. The ROC curve displayed that NHR (AUC = 0.755) and NAR (AUC = 0.756) had moderate predictive performance for amputation in DFU, which was not observed in previous study. XGBoost was selected as the optimal model to predict the risk of amputation in DFU (AUC = 0.894). SHAP analysis revealed that the Wagner degree, LWR, HALP, monocyte, white blood cells and NHR were the primary predictors in the model.

**Conclusion:**

Ten inflammation and nutrition-derived indicators demonstrated significant correlations with amputation. The XGBoost model could predict the risk of amputation in DFU, based on inflammation and nutrition-derived biomarkers (NHR, LWR, and HALP). It may help identify patients at high risk of amputation at an early stage, which could facilitate timely interventions and potentially lower the risk of amputation.

## 1 Introduction

Diabetic foot ulcer (DFU) is one of the most serious complications of diabetes, with approximately 18.6 million people worldwide affected by DFU each year (1). It not only reduces patients’ quality of life and brings financial and psychological burdens but also places substantial fiscal pressure on the healthcare system.

Diabetes-related complications, such as diabetic peripheral neuropathy and peripheral vascular disease, result in the loss of protective sensation and circulatory disorders in the lower extremities, which will increase the risk of foot injuries and delay the healing of wounds (2, 3). Severe foot ulcers can lead to osteomyelitis or even gangrene, posing a risk of amputation (4, 5). The mortality rate is increasing after amputation, with the 5-year mortality rate for patients with DFU standing at approximately 30% (1). Therefore, it is necessary to develop a predictive model for amputation in DFU and identify risk factors of amputation. Previously, the prediction of amputation for DFU were predominantly based on DFU classification systems, such as the University of Texas Diabetic Wound Classification and Wagner Ulcer Classification (6). However, these classification systems mainly rely on clinical experience and fail to provide a thorough evaluation of the impact of demographic data, laboratory biochemical indicators, and other risk factors on amputation.

Inflammatory responses can influence the prognosis of DFU, and inflammatory biomarkers are helpful in predicting the outcomes of DFU (7, 8). It was found that elevated neutrophil-lymphocyte ratio (NLR), increased platelet-lymphocyte ratio (PLR), and decreased lymphocyte-white blood cell ratio (LWR) were associated with increased risk of amputation in DFU (9). Besides, nutritional status also affects the prognosis of DFU. Malnutrition can restrict the proliferation of fibroblasts, limit the synthesis of collagen, and thus be detrimental to the healing of foot wounds (10). It was shown that the neutrophil-albumin ratio (NAR) was positively correlated with the prevalence of DFU (11). But the impact of these nutrition-related biomarkers on DFU is still unclear. At present, there is still a lack of a comprehensive model that predicts amputation in DFU by integrating various inflammation and nutrition-derived indicators.

Machine learning (ML), a branch of artificial intelligence, generates predictive algorithms from large volumes of clinical data and is widely applied in disease diagnosis and prognosis (12, 13). There were some researches focused on the prediction of DFU amputation by using ML algorithms. However, most of the studies had relatively small sample sizes (14–16), resulting in a lack of convincing evidence. And some predictive models relied on professional indicators, such as the Charlson Comorbidity Index (CCI), the foot Infection (WIfI) Classification, and vascular media calcification, which required evaluation by experienced specialists (15, 17, 18), and might hinder their widespread adoption in primary healthcare settings.

This study aims to systematically analyze the predictive value of various inflammatory and nutritional biomarkers (such as NLR, PLR, and NAR) for predicting the risk of amputation in DFU based on the clinical data during hospitalization. Meanwhile, we intended to develop ML models and determine the optimal model for predicting amputation risk based on inflammatory and nutritional markers combined with other clinical data.

## 2 Methods

### 2.1 Study population and data collection

**Figure 1:**
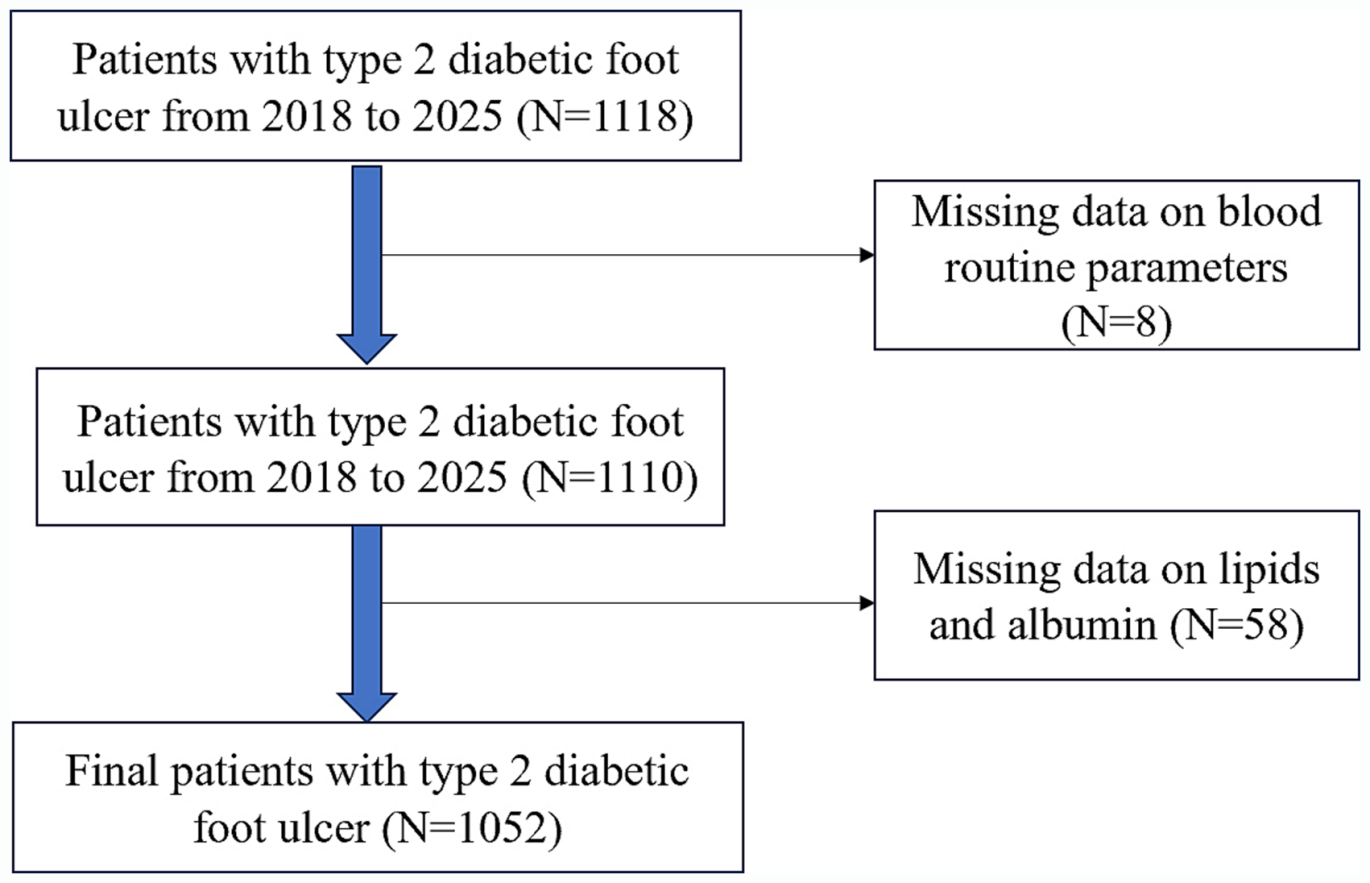
The flowchart of patient selection.

Patients with type 2 diabetic foot ulcer who were hospitalized in the People’s Hospital of Guangxi Zhuang Autonomous Region were selected from January 2018 to December 2025. The inclusion criteria were as follows: (1) Diagnosed with type 2 diabetes according to the guidelines of the American Diabetes Association (ADA) (19). (2) Diagnosed with DFU (Wagner degree 1–5). DFU was defined as foot ulceration or tissue damage, with a defect of all layers of skin (9). The exclusion criteria included: (1) patients younger than 18 years old; (2) patients diagnosed with diabetes types other than type 2 diabetes; (3) patients diagnosed with malignant tumors; (4) patients with missing data on inflammatory and nutritional biomarkers. A total of 1,118 patients with type 2 diabetic foot ulcer were initially included. We excluded patients with missing data on blood routine parameters (*n* = 8), lipids and albumin (*n* = 58), which precluded the calculation of inflammation and nutrition-derived biomarkers. Finally, our study comprised the remaining 1,052 patients. Sample size was estimated via G*Power 3.1 based on previous research (*α* = 0.05, power = 0.90). An additional 10% samples were reserved for potential missing data. The patient selection procedures were presented in Figure 1. Baseline comparisons were conducted between the included and excluded patients. As shown in Supplementary Table S1, no systematic differences in age, gender, Wagner degree, WBC, creatinine, uric acid, albumin and HbA1c were found between the two groups (*p* > 0.05). It suggested that the excluded patients did not result in obvious selection bias. The outcome was amputation within 3 months after admission, encompassing both above-ankle and below-ankle amputations. Some patients requiring amputation also presented with lower extremity arterial stenosis or occlusion and required endovascular intervention first. Amputation surgery was performed during a readmission after improvement of vascular conditions. Therefore, we defined amputation within 3 months as the outcome. The ethics committee of the People’s Hospital of Guangxi Zhuang Autonomous Region approved our study and exempted the need for informed consent, as all analyses were performed on de-identified database records (Approval Number: [2026]017).

We collected the general clinical data and biochemical indicators by reviewing electronic medical records. The general clinical data included gender, age, duration of diabetes, duration of diabetic foot ulcer, Wagner grade (1–5), smoking status, alcohol consumption, history of hypertension, history of coronary heart disease (CHD), history of cerebral infarction, lower extremity arterial disease (PAD, including stenosis or occlusion), diabetic nephropathy, and diabetic peripheral neuropathy. The clinical biochemical indicators encompassed white blood cells (WBC), neutrophils (N), lymphocytes (L), monocytes (M), hemoglobin (Hb), platelets (PLT), creatinine, uric acid, albumin (ALB), alanine aminotransferase (ALT), aspartate aminotransferase (AST), total cholesterol, triglycerides (TG), low-density lipoprotein cholesterol (LDL-C), high-density lipoprotein cholesterol (HDL-C), and glycated hemoglobin (HbA1c). The missing rates of the variables were less than 10%, and multiple imputation was adopted to address the missing data. Based on the above biochemical indicators, the following inflammation and nutrition-related biomarkers were calculated: SIRI = (N × M/L), NLR = N/L, PNR = P/N, NMR = N/M, PLR = P/L, NHR = N/HDL-C, MHR = M/HDL-C, LHR = L/HDL-C, PHR = P/HDL-C, LWR = L/WBC, NAR = N/ALB, HALP=Hb × ALB×L/PLT, Prognostic Nutritional Index (PNI) = ALB+5 × L.

### 2.2 Statistical analysis

The normality of continuous variables was assessed by Shapiro–Wilk test. Normally distributed continuous variables were expressed as mean ± standard deviation and compared by using independent sample t-tests, while non-normally distributed continuous variables were presented as median and interquartile range and compared using Mann–Whitney U tests. Categorical variables were described as frequencies and percentages and compared using chi-square tests or Fisher’s exact tests, as appropriate. Independent predictors for amputation in diabetic foot ulcers were analyzed through multivariate logistic regression analysis. Model 1 was unadjusted. Model 2 was adjusted for age and gender. Model 3 was further adjusted for Wagner degree, smoking, CHD, PAD, Hb, creatinine, uric acid, albumin, LDL-C, HDL-C and HbA1c. The covariates were selected by clinical implication and the results of univariate analysis (Supplementary Table S2). The variance inflation factor (VIF) was used to assess multicollinearity among variables. VIF value less than 5 indicated no obvious multicollinearity. For logistic regression models, ORs were calculated per original unit increase. Standardized ORs (sORs) were calculated per one-standard-deviation increase after standardization. The relationship between inflammation and nutrition-related indexes and amputation was explored using restricted cubic splines (RCS). We used 3 knots (25th, 50th, and 75th) in RCS analysis. The reference value was set at the median. Receiver operating characteristic (ROC) curves were utilized to evaluate the predictive value of derived indicators with the risk of amputation. Delong’s test was adopted to compare AUC differences between different indicators. A *p*-value < 0.05 was considered statistically significant. All statistical analyses were performed using SPSS 22.0, R Studio version 4.3.2, and Python 3.14.2.

### 2.3 Feature selection

The study population was randomly divided, with 80% assigned to the training set and 20% to the validation set. We employed the Least Absolute Shrinkage and Selection Operator (Lasso) regression analysis to select relevant clinical features of amputation from the training set. L1-regularized logistic regression was adopted for feature selection to avoid overfitting and to screen out discriminative features. The estimated coefficients were regularized to approach zero, with the extent of shrinkage controlled by an additional parameter, referred to as *λ*. We utilized 10-fold cross-validation to determine the optimal parameters of the Lasso regression model. When λ = λ.min, the model exhibited the minimum cross-validation error, which offered the most accurate predictions. We chose λ.min as the optimal parameter for the model in order to select the best predictive variables.

### 2.4 Machine learning models and evaluation

Five ML models including extreme gradient boosting (XGBoost), support vector machine (SVM), random forest (RF), light gradient boosting machine (LightGBM), and logistic regression, were developed to predict amputation in patients with DFU. Hyperparameter optimization for all models was performed via grid search combined with 10-fold stratified cross-validation. Multiple imputation was used to impute missing covariates. The following strategies were adopted to address class imbalance. Firstly, the class_weight parameter was set to ‘balanced’ for logistic regression and SVM. For XGBoost and LightGBM, scale_pos_weight was defined as the ratio of negative samples to positive samples. Random forest was trained using both None and balanced class weights. We constructed the receiver operating characteristic (ROC) curve, calibration curve, and decision curve analysis (DCA) curve using the validation set. The predictive performance of five ML models was evaluated by calculating the area under the curve (AUC), accuracy, sensitivity, specificity, negative predictive value (NPV), positive predictive value (PPV), Brier score, F1 score, calibration slope, and calibration intercept on the validation set. F1 score is an indicator that comprehensively evaluate both accuracy and sensitivity (20). Brier score was applied to evaluate the calibration effectiveness. Delong’s test was utilized to compare AUC differences between different models. The optimal model was chosen based on the above evaluation indicators. To improve the accuracy of predicted probabilities, Platt calibration (sigmoid calibration) integrated with 10-fold cross-validation was performed on the model via CalibratedClassifierCV for probability calibration.

### 2.5 Model explanation

The SHapley Additive exPlanations (SHAP) algorithm stands out as a widely favored approach for interpreting model outputs, significantly improving our comprehension of predictions (21). We used SHAP method to assess the contribution of each feature to the prediction of amputation in the optimal model.

## 3 Results

### 3.1 Clinical characteristics of patients with diabetic foot ulcers

In total, we included 1,052 patients with DFU who were hospitalized at the People’s Hospital of Guangxi Zhuang Autonomous Region from January 2018 to December 2025. Among them, 739 patients received conservative treatment and 313 patients underwent amputation within 90 days, including 259 patients who received minor amputation below the ankle, and 54 patients who received major amputation above the ankle. As shown in Table 1, patients who underwent amputation were younger, had a higher Wagner classification degree, and a higher proportion of smokers and peripheral arterial diseases (PAD). Besides, WBC, PLT, creatinine, and HbA1c were higher in patients with amputation than those in non-amputees, while Hb, uric acid, albumin, total cholesterol, LDL-C and HDL-C were lower. In addition, some inflammatory and nutrition indicators in amputation group were increased, including SIRI, NLR, PLR, NHR, MHR, PHR, LHR, NMR, and NAR. However, PNR, LWR, HALP, and PNI were decreased in amputation group.

**Table 1:** Baseline characteristics of patients with diabetic foot ulcers.

Variable	Non-amputation (*n* = 739)	Amputation (*n* = 313)	*p* value
Age (years)	64.7 ± 12.34	62.37 ± 11	0.002
Gender (male), n (%)	516 (69.82%)	237 (75.72%)	0.062
Duration of diabetes (years)	10 (4, 15)	10 (5, 12.22)	0.668
Duration of diabetic foot ulcer (days)	20 (10, 60)	30 (10, 60)	0.011
Wagner degree, n (%)			<0.001
I	24 (3.25%)	0 (0%)	
II	291 (39.38%)	4 (1.28%)	
III	351 (47.5%)	140 (44.73%)	
IV	73 (9.88%)	168 (53.67%)	
V	0 (0%)	1 (0.32%)	
Smoking, n (%)	132 (17.86%)	75 (23.96%)	0.029
Hypertension, n (%)	437 (59.13%)	177 (56.55%)	0.503
CHD, n (%)	127 (17.19%)	33 (10.54%)	0.008
Cerebral infarction, n (%)	117(18.2%)	47(17.1%)	0.682
Diabetic nephropathy, n (%)	356 (48.17%)	130 (41.53%)	0.057
Diabetic peripheral neuropathy, n (%)	586 (79.3%)	245 (78.27%)	0.084
PAD, n (%)	245 (33.15%)	144 (46.01%)	< 0.001
WBC (10^9/L)	8.26 (6.73, 10.37)	11.69 (9.17, 15.86)	< 0.001
N (10^9/L)	5.43 (4.2, 7.62)	8.86 (6.4, 13.13)	< 0.001
L (10^9/L)	1.63 (1.26, 2.07)	1.48 (1.07, 1.95)	0.002
M (10^9/L)	0.64 (0.48, 0.83)	0.85 (0.66, 1.13)	< 0.001
Hb (g/L)	116 (100, 130)	107 (91, 122)	< 0.001
PLT (10^9/L)	271 (221, 352)	344 (279, 421)	< 0.001
SIRI	2.09 (1.28, 3.89)	4.93 (2.64, 10.69)	< 0.001
NLR	3.39 (2.3, 5.3)	6.28 (3.67, 10.53)	< 0.001
PLR	167.83 (123.37, 239.22)	235.53 (172.61, 329.19)	< 0.001
PNR	48.78 (35.43, 65.45)	37.79 (25.76, 54.08)	< 0.001
NHR	5.63 (3.95, 8.64)	10.69 (7.04, 17.85)	< 0.001
MHR	0.65 (0.46, 0.93)	1.01 (0.73, 1.61)	< 0.001
PHR	276.53 (203.92, 400)	423.17 (304.04, 596.2)	< 0.001
LHR	1.68 (1.2, 2.32)	1.95 (1.32, 2.65)	< 0.001
NMR	9.15 (6.96, 11.78)	10.49 (8.04, 13.58)	< 0.001
LWR	0.2 (0.14, 0.26)	0.12 (0.08, 0.19)	< 0.001
NAR	0.16 (0.12, 0.24)	0.29 (0.19, 0.46)	< 0.001
HALP	23.38 (13.29, 36.48)	13.39 (7.9, 22.44)	< 0.001
PNI	42.55 ± 7.11	38.39 ± 7.18	< 0.001
Creatinine (mmol/L)	91 (69, 126)	97 (69.05, 159)	0.017
Uric acid(umol/L)	341 (275, 430)	313 (238, 397.2)	0.001
Albumin (g/L)	34.4 (30.7, 38)	30.8 (26.45, 35)	< 0.001
ALT (U/L)	15 (10, 23)	15.4 (10, 25)	0.489
AST (U/L)	19 (15, 24)	18 (14, 26)	0.563
T-CHOL (mmol/L)	4.16 (3.29, 4.93)	3.68 (3.02, 4.53)	< 0.001
TG (mmol/L)	1.2 (0.9, 1.73)	1.16 (0.87, 1.63)	0.244
LDL-C (mmol/L)	2.66 (2.07, 3.29)	2.33 (1.85, 2.98)	< 0.001
HDL-C (mmol/L)	0.96 (0.78, 1.13)	0.82 (0.65, 0.99)	< 0.001
HbA1c (%)	8.5 (6.9, 10.7)	9.11 (7.5, 11.4)	< 0.001

Data are shown as means ± SD or medians (IQRs) or frequency (percentage). *p* < 0.05 is considered statistically significant. CHD, Coronary heart disease; WBC, White blood cells; N, Neutrophil; L, Lymphocyte; M, Monocyte; Hb, hemoglobin; PLT, Platelet; SIRI, Systemic inflammation response index; NLR, neutrophil-lymphocyte ratio; PLR, Platelet-lymphocyte ratio; PNR, Platelet–neutrophil ratio; NHR, Neutrophil-high-density lipoprotein ratio; MHR, monocyte-high-density lipoprotein ratio; PHR, Platelet-high-density lipoprotein ratio; LHR, Lymphocyte-high-density lipoprotein ratio; NMR, Neutrophil-monocyte ratio; LWR, Lymphocyte-white blood cell ratio; NAR, Neutrophil-albumin ratio; HALP, The hemoglobin, albumin, lymphocyte, platelet score; ALT, Alanine aminotransferase; AST, Aspartate aminotransferase; T-CHOL, Total cholesterol; TG, Triglycerides; LDL-C, Low-density lipoprotein cholesterol; HDL-C, High-density lipoprotein cholesterol; HbA1c, Glycated hemoglobin; PNI, Prognostic nutritional index.

### 3.2 Logistic regression analysis of inflammation and nutrition-derived biomarkers associated with amputation in DFU

Univariate logistic regression analysis revealed that 9 inflammation and nutrition-derived biomarkers were correlated with higher amputation risk, while 3 biomarkers were linked to lower risk (Table 2). After adjusted for corresponding covariates, the multivariate logistic regression analysis showed that 7 biomarkers were positively associated with amputation risk, including SIRI (OR = 1.053, 95% CI: 1.03–1.076, *p* < 0.001), NLR (OR = 1.067, 95% CI: 1.037–1.097, *p* < 0.001), PLR (OR = 1.002, 95% CI: 1.001–1.003, *p* = 0.006), NHR (OR = 1.046, 95% CI: 1.028–1.064, *p* < 0.001), MHR (OR = 1.92, 95% CI: 1.489–2.477, *p* < 0.001), PHR (OR = 1.001, 95% CI: 1.001–1.002, *p* < 0.001), NAR (OR = 7.559, 95% CI: 3.453–16.546, *p* < 0.001). Besides, PNR (OR = 0.99, 95% CI: 0.983–0.997, *p* = 0.005), LWR (OR = 0.008, 95% CI: 0.001–0.073, *p* > 0.001), and HALP (OR = 0.974, 95% CI: 0.962–0.986, *p* > 0.001) were negatively correlated with amputation (Table 2). OR was calculated per original unit increase. VIF between the variables were less than 5, which suggested no obvious multicollinearity among variables (Supplementary Table S3). However, LHR, NMR and PNI were not independently related to the risk of amputation (*p* > 0.05). Since ORs for indicators such as NAR and LWR might be strongly affected by scale, we adopted standardized ORs to compare the effect sizes across different indicators. After adjusted for corresponding covariates, MHR showed the highest OR per one-standard-deviation increase after standardization (Table 3). Given that patients with missing data on inflammation and nutrition-derived indicators were excluded during participant selection, we further applied multiple imputation to minimize bias caused by missing values. We conducted a sensitivity analysis to compare the results between complete-case analysis and imputation-based analysis. It indicated that elevated levels of SIRI, NLR, PLR, NHR, MHR, PHR and NAR were associated with increased risk of amputation, while PNR, LWR and HALP were negatively correlated with amputation in imputation-based analysis (Supplementary Table S4), which was consistent with the results of complete-case analysis. These findings confirmed the robustness of our conclusions.

**Table 2:** Logistic regression analysis of factors associated with amputation risk of diabetic foot ulcers.

variables	Model 1		Model 2		Model 3	
	OR (95% CI)	*p* value	OR (95% CI)	*p* value	OR (95% CI)	*p* value
SIRI	1.099 (1.074,1.125)	< 0.001	1.099 (1.074,1.125)	< 0.001	1.053 (1.03,1.076)	< 0.001
NLR	1.125 (1.094,1.156)	< 0.001	1.123 (1.093,1.154)	< 0.001	1.067 (1.037,1.097)	< 0.001
PLR	1.004 (1.003,1.005)	< 0.001	1.004 (1.003,1.005)	< 0.001	1.002 (1.001,1.003)	0.006
PNR	0.98 (0.973,0.986)	< 0.001	0.98 (0.974,0.986)	< 0.001	0.99 (0.983,0.997)	0.005
NHR	1.091(1.071,1.111)	< 0.001	1.091(1.071,1.111)	< 0.001	1.046 (1.028,1.064)	< 0.001
MHR	3.294 (2.591,4.187)	< 0.001	3.294 (2.591,4.187)	< 0.001	1.92 (1.489,2.477)	< 0.001
PHR	1.003(1.002,1.003)	< 0.001	1.003(1.002,1.003)	< 0.001	1.001 (1.001,1.002)	< 0.001
LHR	1.257 (1.1,1.436)	0.001	1.213 (1.058, 1.391)	0.006		0.831
NMR	1.049 (1.025,1.074)	< 0.001	1.047 (1.022, 1.072)	< 0.001		0.24
LWR	< 0.001 (< 0.001, < 0.001)	< 0.001	< 0.001 (< 0.001, 0.001)	< 0.001	0.008 (0.001, 0.073)	< 0.001
NAR	50.333(22.919, 110.54)	< 0.001	50.333(22.919, 110.54)	< 0.001	7.559 (3.453,16.546)	< 0.001
HALP	0.949 (0.938,0.96)	< 0.001	0.95 (0.939,0.961)	< 0.001	0.974 (0.962,0.986)	< 0.001
PNI	0.922 (0.904, 0.94)	< 0.001	0.924 (0.906, 0.942)	< 0.001		0.224

Model 1: Unadjusted; Model 2: Adjusted for age and gender; Model 3: Adjusted for age, gender, Wagner degree, smoking, CHD, PAD, Hb, creatinine, uric acid, albumin, LDL-C, HDL-C and HbA1c. P < 0.05 is considered statistically significant. OR, Odds ratio per original unit increase. SIRI, Systemic inflammation response index; NLR, Neutrophil-lymphocyte ratio; PLR, Platelet-lymphocyte ratio; PNR, Platelet–neutrophil ratio; NHR, Neutrophil-high-density lipoprotein ratio; MHR, Monocyte-high-density lipoprotein ratio; PHR, Platelet-high-density lipoprotein ratio; LHR, Lymphocyte-high-density lipoprotein ratio; NMR, Neutrophil-monocyte ratio; LWR, Lymphocyte-white blood cell ratio; NAR, Neutrophil-albumin ratio; HALP, The hemoglobin, albumin, lymphocyte, platelet score; PNI, Prognostic nutritional index.

**Table 3:** Logistic regression analysis of factors associated with amputation risk of diabetic foot ulcers using standardized odds ratios.

Variables	Model 1		Model 2		Model 3	
	sOR (95% CI)	*p* value	sOR (95% CI)	*p* value	sOR (95% CI)	*p* value
SIRI	2.147 (1.78, 2.589)	< 0.001	2.147 (1.78, 2.589)	< 0.001	1.517 (1.271, 1.811)	< 0.001
NLR	2.008 (1.709, 2.36)	< 0.001	1.988 (1.691, 2.338)	< 0.001	1.466 (1.238, 1.737)	< 0.001
PLR	1.66 (1.448,1.903)	< 0.001	1.659 (1.447, 1.902)	< 0.001	1.255 (1.068, 1.475)	0.006
PNR	0.606 (0.517,0.712)	< 0.001	0.61 (0.52, 0.715)	< 0.001	0.777 (0.652, 0.927)	0.005
NHR	2.504 (2.065, 3.038)	< 0.001	2.504 (2.065, 3.038)	< 0.001	1.613 (1.345, 1.933)	< 0.001
MHR	2.525 (2.096, 3.043)	< 0.001	2.525 (2.096, 3.043)	< 0.001	1.66 (1.363, 2.023)	< 0.001
PHR	2.15 (1.813, 2.55)	< 0.001	2.15 (1.813, 2.55)	< 0.001	1.43 (1.193, 1.715)	< 0.001
LHR	1.25 (1.098,1.423)	0.001	1.207 (1.056, 1.379)	0.006		0.831
NMR	1.35 (1.164,1.565)	< 0.001	1.33 (1.147, 1.542)	< 0.001		0.24
LWR	0.426 (0.361, 0.502)	< 0.001	0.428 (0.363, 0.504)	< 0.001	0.603 (0.503, 0.721)	< 0.001
NAR	2.324 (1.962, 2.752)	< 0.001	2.324 (1.962, 2.752)	< 0.001	1.545 (1.306, 1.829)	< 0.001
HALP	0.357 (0.283,0.449)	< 0.001	0.363 (0.289, 0.457)	< 0.001	0.595 (0.463, 0.764)	< 0.001
PNI	0.549 (0.475, 0.635)	< 0.001	0.557 (0.482, 0.644)	< 0.001		0.225

Model 1: Unadjusted; Model 2: Adjusted for age and gender; Model 3: Adjusted for age, gender, Wagner degree, smoking, CHD, PAD, Hb, creatinine, uric acid, albumin, LDL-C, HDL-C and HbA1c. P < 0.05 is considered statistically significant. sOR: standardized odds ratio per one-standard-deviation increase after standardization. SIRI: systemic inflammation response index; NLR: neutrophil-lymphocyte ratio; PLR: platelet-lymphocyte ratio; PNR: platelet–neutrophil ratio; NHR: neutrophil-high-density lipoprotein ratio; MHR: monocyte-high-density lipoprotein ratio; PHR: platelet-high-density lipoprotein ratio; LHR: lymphocyte-high-density lipoprotein ratio; NMR: neutrophil-monocyte ratio; LWR: lymphocyte-white blood cell ratio; NAR: neutrophil-albumin ratio; HALP: the hemoglobin, albumin, lymphocyte, platelet score; PNI: prognostic nutritional index.

**Figure 2:**
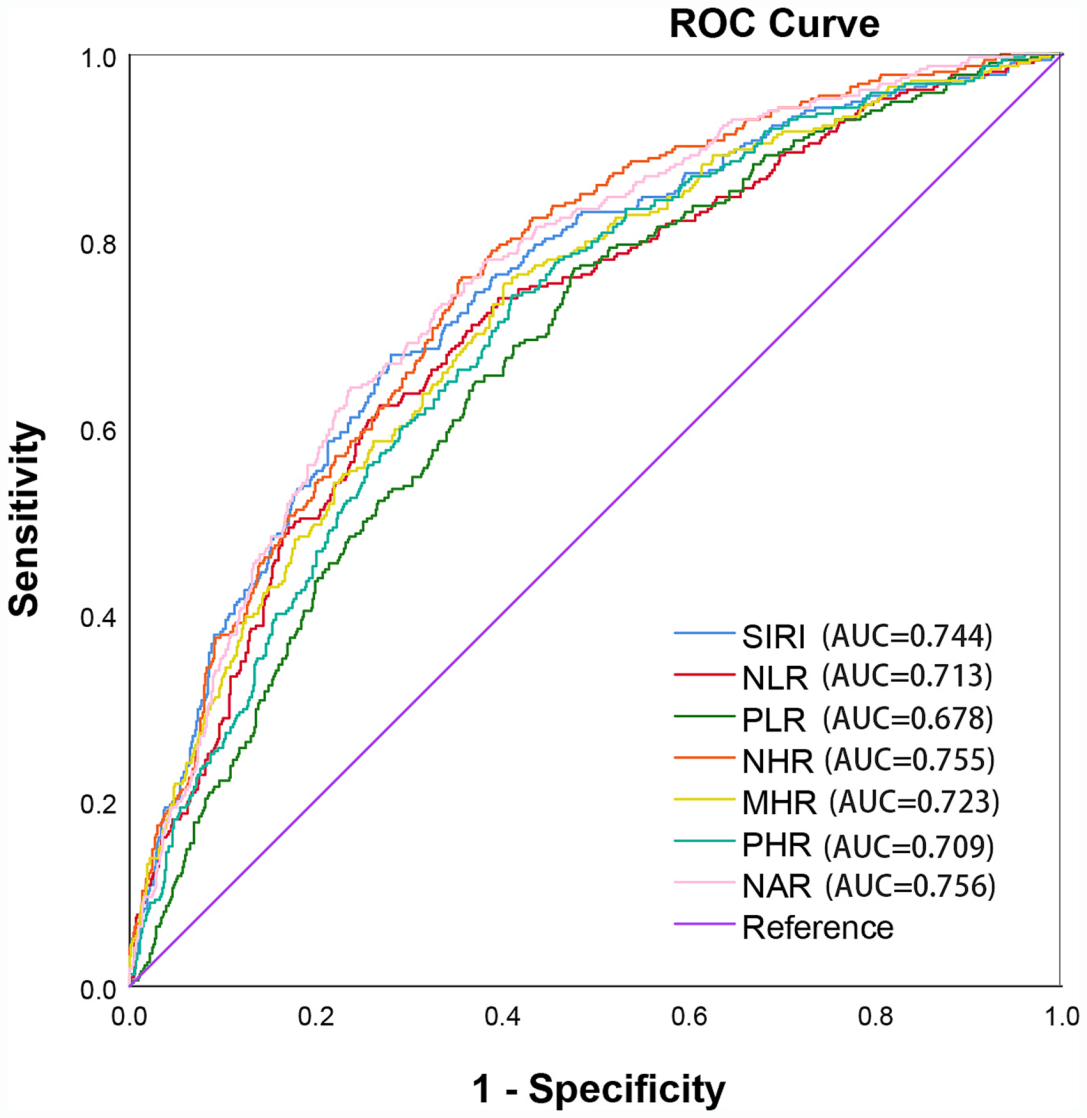
ROC analysis to determine the diagnostic performance of inflammation and nutrition-derived biomarkers in predicting amputation in diabetic foot ulcers. SIRI, Systemic inflammation response index; NLR, Neutrophil-lymphocyte ratio; PLR, Platelet-lymphocyte ratio; NHR, Neutrophil-high-density lipoprotein ratio; MHR, Monocyte-high-density lipoprotein ratio; PHR, Platelet-high-density lipoprotein ratio; NAR, Neutrophil-albumin ratio.

ROC curves were employed to evaluate the predictive ability of biomarkers for amputation. As shown in Figure 2, the ROC curves of biomarkers with AUC>0.5 were presented. The Delong’s test revealed that NAR (AUC = 0.756) exhibited better discrimination capacity than NLR (AUC = 0.713, *p* < 0.001), PLR (AUC = 0.678, *p* < 0.001), MHR (AUC = 0.723, *p* = 0.028), and PHR (AUC = 0.709, *p* = 0.007), while no significant difference was observed when compared with SIRI (AUC = 0.744, *p* = 0.198) and NHR (AUC = 0.755, *p* = 0.934) (Supplementary Table S5).

### 3.3 RCS analysis of the relationship between inflammation and nutrition-derived biomarkers and amputation risk in DFU

**Figure 3:**
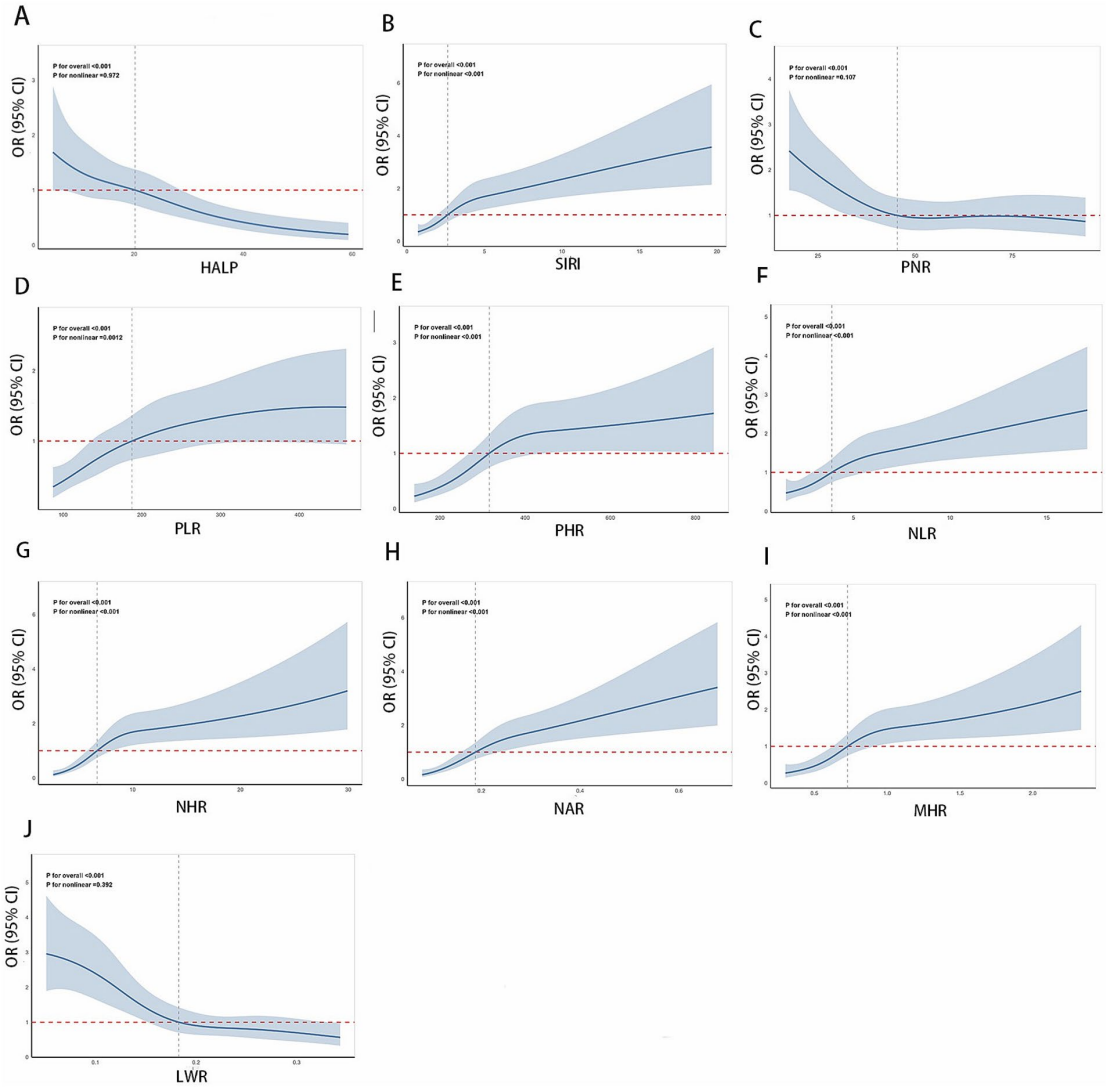
The relationship between inflammation and nutrition-derived biomarkers and amputation in diabetic foot ulcers by using restricted cubic splines (RCS). **(A)** HALP; **(B)** SIRI; **(C)** PNR; **(D)** PLR; **(E)** PHR; **(F)** NLR; **(G)** NHR; **(H)** NAR; **(I)** MHR; **(J)** LWR. Abbreviations: SIRI: systemic inflammation response index; NLR: neutrophil-lymphocyte ratio; PLR: platelet-lymphocyte ratio; PNR: platelet-neutrophil ratio; NHR: neutrophil-high-density lipoprotein ratio; MHR: monocyte-high-density lipoprotein ratio; PHR: platelet-high-density lipoprotein ratio; LWR: lymphocyte-white blood cell ratio; NAR: neutrophil-albumin ratio; HALP: the hemoglobin, albumin, lymphocyte, platelet score.

The RCS analysis indicated that NHR (*p* for overall < 0.001, *p* for nonlinear < 0.001), MHR (*p* for overall < 0.001, *p* for nonlinear < 0.001), PHR (P for overall < 0.001, *p* for nonlinear < 0.001), NAR (*p* for overall < 0.001, *p* for nonlinear < 0.001), NLR (*p* for overall < 0.001, *p* for nonlinear < 0.001), PLR (*p* for overall < 0.001, *p* for nonlinear = 0.0012), and SIRI (*p* for overall < 0.001, *p* for nonlinear < 0.001) had nonlinear and positive relationships with the risk of amputation after adjusting for age, gender, smoking, CHD, PAD, Hb, creatinine, uric acid, albumin, LDL-C, HDL-C and HbA1c. These findings indicated that the amputation rate increased rapidly with increasing inflammatory response. Besides, HALP (*p* for overall < 0.001, *p* for nonlinear = 0.972), LWR (*p* for overall < 0.001, *p* for nonlinear = 0.392), and PNR (*p* for overall < 0.001, *p* for nonlinear = 0.107) showed linear and negative associations with amputation (Figure 3).

### 3.4 Subgroup analysis to analyze the association between inflammation and nutrition-derived indicators and amputation in DFU among different age groups

The associations between different inflammation and nutrition-derived indicators and amputation in DFU were analyzed across different age groups. As shown in Table 4, significant interaction was discovered between PLR, PNR, LWR, HALP and amputation in different age subgroups (*p* < 0.05 for interaction). Higher levels of PLR and lower levels of PNR, LWR, and HALP were related to increased risk of amputation among patients whose age ≥ 60 (*p* < 0.05). However, no significant correlation was observed between PLR, PNR, HALP and amputation among patients whose age < 60 (*p* > 0.05). Besides, the association between LWR and amputation was stronger in elderly patients (age ≥ 60) than younger patients (age < 60). It was found that age had no significant impact on the correlation between SIRI, NLR, NHR, MHR, PHR, and NAR and amputation (*p* > 0.05 for interaction).

**Table 4:** Age-stratified subgroup analysis to evaluate the association between inflammation and nutrition-derived indicators and amputation in DFU.

Characteristic	OR (95% CI)	*p* value	*p* for interaction
SIRI			0.559
Age < 60	1.039 (1.009, 1.07)	0.009	
Age ≥ 60	1.065 (1.03, 1.1)	< 0.001	
NLR			0.46
Age < 60	1.046 (1.007, 1.086)	0.02	
Age ≥ 60	1.083 (1.04, 1.128)	< 0.001	
PLR			0.045
Age < 60		0.169	
Age ≥ 60	1.002 (1.000, 1.004)	0.011	
PNR			< 0.001
Age < 60		0.201	
Age ≥ 60	0.985 (0.976, 0.995)	0.002	
NHR			0.949
Age < 60	1.035 (1.012, 1.058)	0.003	
Age ≥ 60	1.055 (1.028, 1.083)	< 0.001	
MHR			0.851
Age < 60	1.634 (1.163, 2.295)	0.005	
Age ≥ 60	2.274 (1.554, 3.327)	< 0.001	
PHR			0.256
Age < 60	1.001 (1.000, 1.002)	0.007	
Age ≥ 60	1.001 (1.000, 1.002)	0.012	
LWR			< 0.001
Age < 60	0.032 (0.002, 0.588)	0.02	
Age ≥ 60	0.001 (< 0.001, 0.011)	< 0.001	
NAR			0.768
Age < 60	4.402 (1.557, 12.444)	0.005	
Age ≥ 60	11.748 (3.648, 37.834)	< 0.001	
HALP			< 0.001
Age < 60		0.197	
Age ≥ 60	0.964 (0.947, 0.982)	< 0.001	

SIRI, Systemic inflammation response index; NLR, Neutrophil-lymphocyte ratio; PLR, Platelet-lymphocyte ratio; PNR, Platelet–neutrophil ratio; NHR, Neutrophil-high-density lipoprotein ratio; MHR, Monocyte-high-density lipoprotein ratio; PHR, Platelet-high-density lipoprotein ratio; LWR, Lymphocyte-white blood cell ratio; NAR, Neutrophil-albumin ratio; HALP, The hemoglobin, albumin, lymphocyte, platelet score.

### 3.5 Machine learning models predicting the risk of amputation in DFU

**Figure 4:**
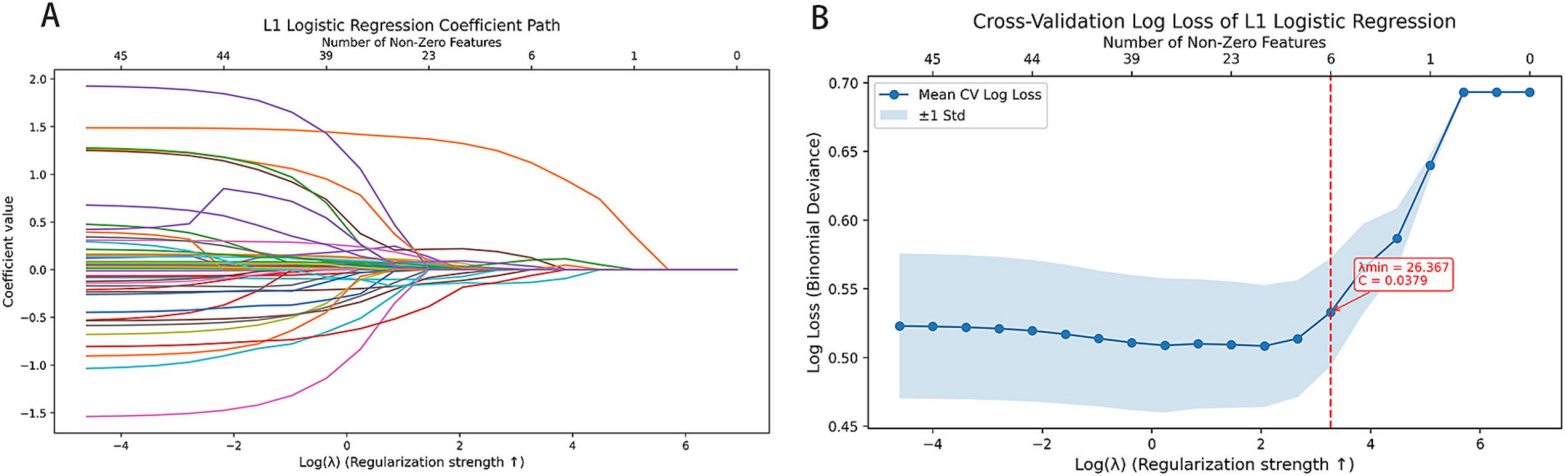
Lasso regression analysis of potential variables selection in prediction model. **(A)** Analysis of clinical features using Lasso regression coefficients. **(B)** The regularization parameter *λ* selection for Lasso regression using 10-fold cross-validation.

**Figure 5:**
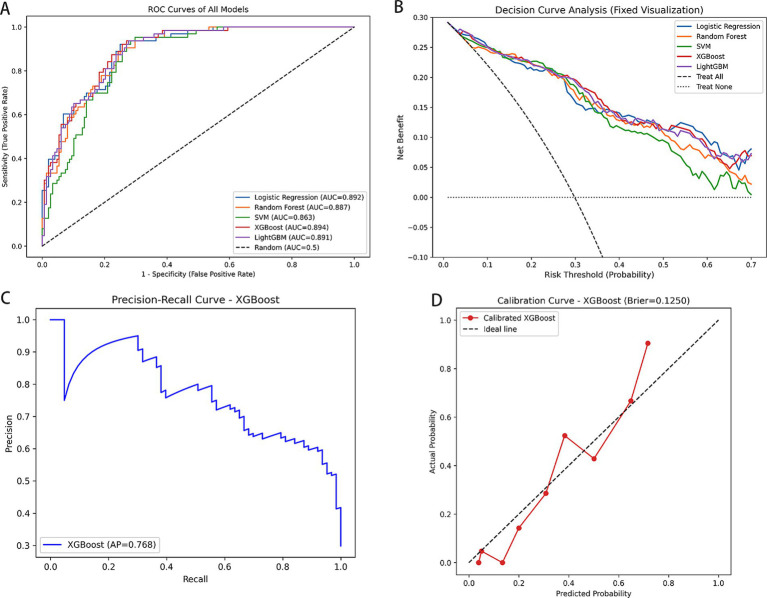
The discrimination and calibration abilities of the machine learning models. **(A)** Receiver operating characteristic (ROC) curves of 5 ML models for predicting amputation risk in diabetic foot ulcers. **(B)** Decision curve analysis (DCA) curves of 5 models. **(C)** Precision-Recall curve of XGBoost model for predicting amputation. **(D)** Calibration curve of XGBoost for predicting amputation. SVM: Support Vector Machine; XGBoost: Extreme Gradient Boosting; LightGBM: Light Gradient Boosting Machine; ROC: Receiver operating characteristic; AUC: area under the curve; AP: average precision.

The study population was divided into a training set and a validation set in a ratio of 8:2. Lasso regression analysis was employed to screen for features associated with amputation from the training set (Figure 4A). We chose *λ*min (λ = 26.367) as the ultimate parameter for conducting the Lasso regression analysis (Figure 4B). Six variables were selected as predictive indicators of amputation, including Wagner degree, WBC, monocyte, NHR, LWR, and HALP. We utilized five ML models to predict the risk of amputation by using logistic regression, random forest, SVM, XGBoost, and LightGBM. The performance metrics of the five models were summarized in Table 5. The AUC (95% CI) of these five ML models were 0.892 (0.845, 0.931), 0.887 (0.835, 0.929), 0.863 (0.814, 0.909), 0.894 (0.849, 0.934) and 0.891 (0.846, 0.931) in the validation set (Figure 5A). Among all models, the XGBoost model yielded the highest AUC, which was significantly higher than SVM (*p* < 0.05) by using Delong’s test (Supplementary Table S6). Although there were no significant differences between XGBoost and logistic regression, random forest and LightGBM in terms of AUC (*p* > 0.05), the XGBoost model still exhibited better discriminative performance with the highest accuracy, F1 score and lower Brier score. Figure 5B displayed the DCA curves in a visual manner, offering a clear demonstration of the model’s practical clinical value. All models provided positive net benefit above the “Treat All” and “Treat None” lines. The Logistic regression, XGBoost and LightGBM models maintained higher and more stable net benefit among all models. In summary, XGBoost was chosen as the optimal model for predicting amputation in DFU. The Precision-Recall curve verified the solid diagnostic accuracy and reliability of XGBoost model (Figure 5C). The calibration curve exhibited a comparatively minor divergence from the ideal curve, with a calibration slope of 1.445 and intercept of 0.167. It suggested acceptable accuracy in predicting the amputation risk in patients with DFU (Figure 5D).

**Table 5:** Performance evaluation of machine learning models.

Model	AUC	Accuracy	Sensitivity	Specificity	NPV	PPV	Brier score	F1 score	calibration slope	calibration intercept
Logistic regression	0.892	0.815	0.619	0.899	0.847	0.722	0.125	0.667	1.306	0.107
Random forest	0.887	0.810	0.556	0.919	0.829	0.745	0.13	0.636	1.344	0.14
SVM	0.864	0.796	0.667	0.851	0.857	0.656	0.138	0.661	1.308	0.092
XGBoost	0.894	0.820	0.619	0.905	0.848	0.736	0.125	0.672	1.445	0.167
LightGBM	0.891	0.815	0.556	0.926	0.83	0.761	0.126	0.642	1.429	0.157

SVM, Support Vector Machine; XGBoost, Extreme Gradient Boosting; LightGBM, Light Gradient Boosting Machine; AUC, Area under the curve; NPV, Negative predictive value; PPV, Positive predictive value.

### 3.6 Model explainability by SHAP

**Figure 6:**
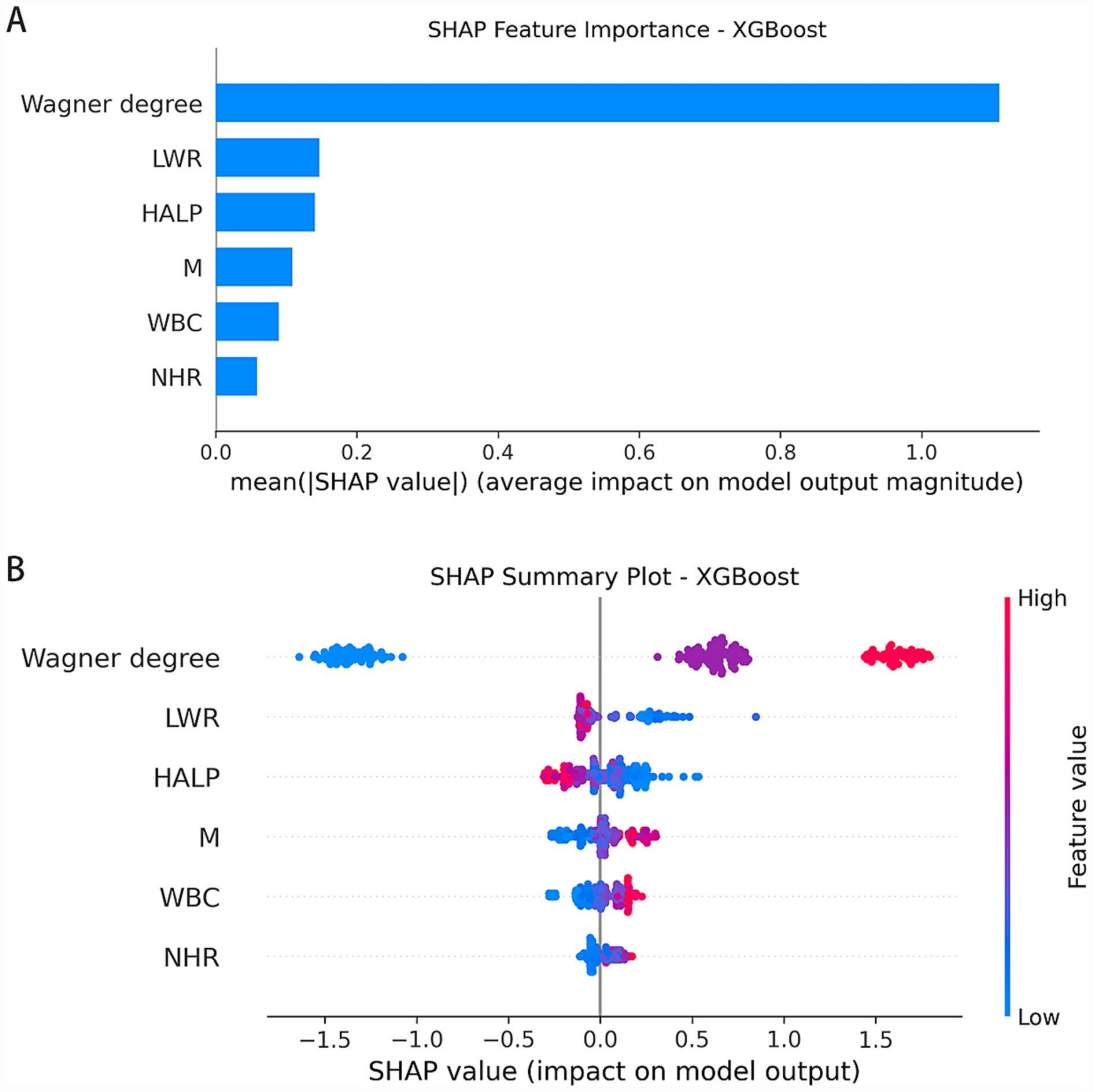
SHAP summary plot of clinical features of XGBoost model. **(A)** Feature importance of amputation model using SHA*p* values. **(B)** Beeswarm plot of the SHAp values for six features in the model. Each dot showed the effect of a feature on the prediction of amputation for one patient. The color gradient from blue to red represents low to high values of the respective feature. Negative SHAp values (left of the x-axis) reflected reduced amputation risk predictions, while positive values (right of the x-axis) reflected increased risk predictions. HALP: the hemoglobin, albumin, lymphocyte, platelet score; LWR: lymphocyte-white blood cell ratio; WBC: white blood cell count; M: monocyte; NHR: neutrophil-high-density lipoprotein ratio.

The influence of each feature on the prediction model was analyzed by calculating the SHAp values, thereby determining which factors contribute the most for predicting amputation in DFU. In the prediction model, the contributions of the six factors for predicting amputation were sorted as Wagner degree, LWR, HALP, monocyte, WBC, and NHR (Figures 6A,B). Elevated levels of Wagner degree, monocytes, WBC, and NHR reflected increased risk of amputation, while elevated levels of LWR and HALP suggested decreased risk of amputation.

**Figure 7:**
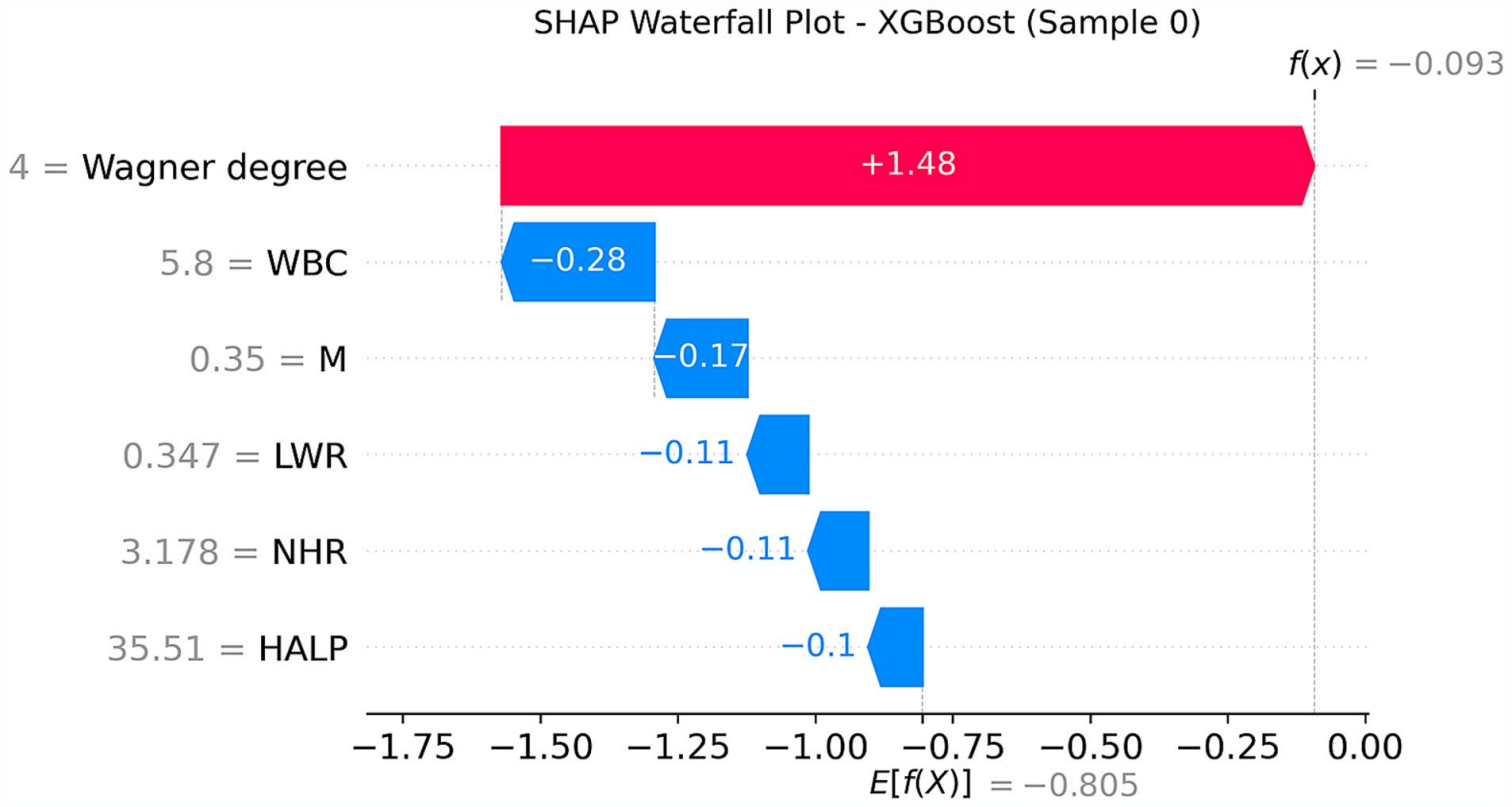
The SHAP waterfall plot illustrated the contribution of all features to the predicted amputation risk for an individual patient in XGBoost model. The SHAp value of each feature was represented by an arrow. Red arrows showed that the corresponding SHAp value increased the model’s prediction value, while blue arrows indicated a negative contribution. The predicted value for an individual (f(X)) was equal to the expected prediction value (E[f(X)]) plus the SHAp values of each feature. HALP: the hemoglobin, albumin, lymphocyte, platelet score; LWR: lymphocyte-white blood cell ratio; WBC: white blood cell count; M: monocyte; NHR: neutrophil-high-density lipoprotein cholesterol ratio.

The SHAP waterfall plot presented the influence of all features on the amputation prediction result for an individual patient by XGBoost, providing a basis for clinicians’ decision-making (Figure 7). The predicted value for an individual was obtained by adding the expected prediction value to the SHAP values of each feature. The baseline amputation risk prediction value for this patient was E[f(X)] = −0.805, which was obtained by calculating the average prediction value of amputation risk in the training set. The patient had a high Wagner degree with its SHAP value of +1.48, significantly increasing the predicted risk of amputation. However, the lower levels of WBC, monocytes, NHR, and relatively high levels of LWR and HALP reduced the predicted risk by −0.28, −0.17, −0.11, −0.11, and −0.1. Ultimately, the predicted risk of amputation for this patient is f(X) = −0.093.

## 4 Discussion

We investigated the association between 13 inflammation and nutrition-derived biomarkers and the amputation risk of DFU patients. After adjusting for confounding factors, 10 indicators were independently associated with the risk of amputation, including SIRI, NLR, PLR, PNR, NHR, MHR, PHR, LWR, NAR, and HALP. Among them, NHR and NAR had better predictive performance for the risk of amputation. In addition, ML models were constructed to predict the risk of amputation, based on the inflammation and nutrition biomarkers, demographic characteristics, and other clinical biochemical indicators. Ultimately, XGBoost was selected as the optimal model, and Wagner degree, WBC, monocyte count, NHR, LWR, and HALP were important predictors in the amputation model. Besides, the SHAP algorithm was applied to display the impact of each clinical feature on the prediction results.

Ten inflammation and nutrition biomarkers were independent determinants of amputation in patients with DFU. RCS analysis indicated that SIRI, NLR, PLR, NHR, MHR, PHR, and NAR were non-linearly positively associated with the risk of amputation, while PNR, LWR, and HALP were linearly negatively correlated with amputation. However, the OR values of SIRI, NLR, PLR, PNR, NHR, PHR, and HALP were around 1.05, suggesting weak influence on amputation and limited clinical utility when used as a standalone predictor. The ROC curves showed that the AUC values for NHR and NAR were higher than other inflammation and nutrition-derived indicators. However, both AUC values were less than 0.8, representing only moderate predictive ability. It suggested that combining multiple markers was required to improve the predictive performance for amputation. Inflammation and nutritional status were important factors that affected the wound healing in DFU. Chronic inflammation and immune dysfunction would damage the healing process of the wound, leading to persistent infection and tissue damage. Pro-inflammatory cytokines such as TNF-*α*, IL-1β, and IL-6 would hinder angiogenesis and delay wound repair (22). Immune cells such as neutrophils, macrophages, and T lymphocytes played crucial roles in the development of inflammation (23). It was found that exhausted regulatory T cells, impaired neutrophil function, and compromised angiogenesis in DFU wound would promote chronic inflammation (24). Besides, malnutrition would prolong the inflammatory period, constrain collagen production, delay wound healing, and increase the risk of developing new wounds (10). Therefore, it is necessary to comprehensively assess the inflammation and nutrition-derived indicators for predicting the prognosis of DFU.

HDL-related inflammatory indicators are novel biomarkers reflecting the inflammatory state and lipid metabolism. HDL has antioxidant and anti-inflammatory effects and can protect against diabetic peripheral artery disease, which might reduce the incidence of DFU (25). It was suggested that NHR, MHR, and PHR were positively correlated with the prevalence of DFU and could be used as biomarkers for predicting DFU (26). Higher levels of NHR indicated increased neutrophils count and activity, and decreased anti-inflammatory ability of HDL (26). Excessive release of pro-inflammatory cytokines and reactive oxygen species by neutrophils would delay wound healing (27). Besides, MHR was also closely associated with chronic inflammatory diseases, such as metabolic syndrome (28) and diabetic nephropathy (29). Monocytes were capable of secreting both pro-inflammatory cytokines and anti-inflammatory cytokines. They could differentiate into macrophages, which served as crucial immune cells that initiate the inflammatory response (27). HDL reduced the pro-inflammatory effect of monocytes by inhibiting the activation of monocyte, as well as the proliferation and differentiation of monocyte progenitor cells (30). PHR reflected platelet-derived inflammation and dysfunction of HDL (26). Platelet activation played important role in DFU through modulating microRNAs via platelet-rich fibrin (31). To our knowledge, the studies on the relationship between HDL-related inflammatory indicators and the risk of amputation in DFU were limited. This study revealed that NHR, MHR, and PHR were independently positively correlated with amputation in DFU, while LHR had no independent correlation with amputation. The ROC curve indicated that NHR exhibited moderate predictive performance for the risk of amputation.

We found that NLR, PLR, PNR, LWR, and SIRI were associated with amputation in patients with DFU. Previous research demonstrated that higher levels of NLR and PLR indicated increased risk of amputation, while elevated LWR decreased the risk of amputation (9). This was consistent with our findings. Elevated NLR would lead to endothelial dysfunction by increasing neutrophil activity (7), which served as an independent indicator of wound healing (32). Higher PLR values indicated a stronger inflammatory response and pro-thrombotic state, which was not conducive to wound healing and increased the risk of amputation (33). Lower LWR values suggested heightened oxidative stress, excessive polarization of M1 macrophages, and defective angiogenesis, which might delay wound healing (34). Another research also discovered that lower LWR indicated a higher risk of amputation (35). Furthermore, our research revealed that SIRI was independently positively associated with amputation in DFU, and PNR was independently negatively correlated with amputation, which was not observed in previous studies. SIRI reflected patients’ immune capability and inflammatory states, based on neutrophils, monocytes, and lymphocytes (36). It was suggested that SIRI was associated with the prevalence of DFU in previous research (36). Besides, lower PNR was associated with increased risk of lower extremity arterial disease in patients with T2DM (37). Lower extremity arterial disease was a risk factor for amputation in DFU (38). Therefore, SIRI and PNR were also important biomarkers for predicting amputation in DFU. Interestingly, we found that there was no significant association between PLR, PNR and amputation in patients younger than 60. Given that elderly patients with DFU might demonstrate different inflammatory responses, nutritional characteristics, comorbidity burden, and amputation risks, further research is required to elucidate the mechanism of inflammation in DFU progression among elderly patients.

Malnutrition led to impaired collagen synthesis and was necessary to wound healing. Therefore, a comprehensive analysis of inflammatory and nutritional indicators was important for assessing the prognosis of DFU. We discovered that NAR was positively related to amputation and HALP reduced the risk of amputation. However, the correlation between HALP and amputation was not significant among patients younger than 60. NAR was the ratio of neutrophils to albumin, reflecting the balance between systemic inflammation and nutritional status (39). The NAR confidence intervals in Model 1 and Model 2 were wide. This was mainly attributed to the small sample size and low event rate of amputation. It would result in a larger deviation in model fitting and a wider confidence interval. Therefore, we should enlarge the sample size and increase the number of amputation events to enhance the stability of statistical results in further research. The HALP score combined albumin, hemoglobin, lymphocyte counts and platelet counts, and comprehensively assesses inflammation and nutritional status (40). Albumin was beneficial for collagen synthesis, cell regeneration and angiogenesis. Hypoproteinemia was detrimental to wound healing and increased the risk of amputation (41). Moreover, anemia led to decreased blood oxygen levels, exacerbated lower limb ischemia and hindered wound healing (42). Previous research discovered that HALP score was a new predictor for major amputation in DFU (43). Another research found that patients with high NAR and low HALP had increased prevalence of DFU (11). To our knowledge, there were currently no studies focused on the correlation between NAR and amputation in DFU. We found that NAR and HALP were independent predictors of amputation in DFU. The ROC curve showed that NAR had a moderate predictive value for amputation. Interestingly, PNI were not independently associated with amputation in our study. PNI was a widely used indicator to assess the nutritional and immune status of patients. Previous research discovered that low levels of PNI was related to increased risk of amputation in DFU (44), which was inconsistent with the results of our study. Future prospective multicenter cohort studies are needed to explore the association and mechanism between PNI and amputation in DFU.

Interestingly, the patients in the amputation group were younger than those in the non-amputation group in our study. The conclusion of the association between age and amputation risk in DFU was inconsistent in previous research. Some studies indicated that patients with older age was associated with delayed healing and higher risk of amputation (45, 46). It was found that age ≥ 70 years was identified as a risk factor for lower-extremity amputation in DFU (46). However, another research reported no significant difference in minor amputation rate between younger and older patients (47). In our study, we discovered that the patients in amputation group were younger than non-amputation group. This discrepancy might be explained by the fact that the outcome of our study included both minor and major amputations. Older patients usually had poorer cardiovascular and pulmonary functions and lower tolerance to major amputation surgery compared to younger patients. Therefore, they tended to receive conservative treatment instead. Further multi-center studies are warranted to explore the association between age and different types of amputation, including minor and major amputations.

Based on the above inflammation and nutrition-derived biomarkers, we employed five ML models to predict the risk of amputation in patients with DFU. XGBoost was chosen as the optimal model. ML models were widely applied in predicting the occurrence and prognosis of various diseases. There were some previous studies established prediction models for amputation in DFU using ML models. A study predicted the risk of non-amputation, minor amputation, and major amputation in DFU by using the LightGBM model, obtaining AUC values of 0.9, 0.85, and 0.86, respectively. However, the study only employed one ML model, which might decrease the reliability and robustness of the conclusion (15). Another research employed eight ML models to predict amputation in DFU. XGBoost was the optimal model, and the AUC values for predicting major amputation, minor amputation, and any amputation risk were 0.82, 0.637, and 0.756, respectively (17). The AUC of this model was relatively low and needs to improve the ability to distinguish high-risk groups for amputation. In our study, we selected features through Lasso regression analysis and constructed prediction model of amputation by employing five ML models, including logistic regression, SVM, XGBoost, LightGBM, and random forest. It enhanced the robustness of the results and was conducive to choose the model with stronger discrimination ability and better clinical utility. Ultimately, XGBoost was selected as the optimal model. XGBoost had attracted great attention due to its advantages of good predictive performance, high computational efficiency, and strong generalization ability (48). We chose Wagner degree, LWR, HALP, NHR, WBC, and monocytes for model construction based on lasso regression results. Wagner degree reflected the severity of foot ulcers, including wound size, depth, and infection degree. LWR, HALP, and NHR were all derived indicators based on routine blood tests, blood lipids, and liver function tests, which could be easily implemented in primary health system. The AUC of the model was 0.894, indicating a good discrimination ability for people at high risk of amputation of DFU. The PR curve and calibration curve also demonstrated satisfactory predictive capability for amputation.

In addition, the SHAP estimation was applied to explain the prediction results of the model, showing the contribution of each feature to the model’s prediction results. According to the results of SHAP analysis, the importance of predictors for amputation were Wagner degree, LWR, HALP, monocytes, WBC, and NHR, ranked from high to low. LWR and HALP were negatively correlated with amputation, while monocytes, WBC, and NHR were positively correlated with amputation. These findings were consistent with the results of multivariate logistic regression analysis, indicating that inflammation could exacerbate tissue damage, delay wound healing, and increase the risk of amputation. And malnutrition restricted collagen synthesis and was harmful for wound healing, including hypoalbuminemia and anemia.

The advantages of the study were as follows: Firstly, it systematically analyzed the correlations of 13 inflammation and nutrition-derived indicators with amputation in DFU, providing new biomarkers for early identification of high-risk groups for amputation. Secondly, we constructed ML models to predict diabetic foot amputation based on inflammation and nutrition-derived biomarkers and DFU classification system (Wagner classification). The features employed in the model were all derived from commonly used clinical biochemical indicators, facilitating its promotion in primary hospitals.

The study also had some limitations. Firstly, this was a single-center study with only internal validation via random data splitting. The lack of external validation restricted the generalizability of the model across different time periods, clinical workflows, hospitals, and patient populations. Further external validation based on multi-center datasets is required before clinical application. Secondly, our study only indicated that NHR, HALP, and other biomarkers were independently associated with amputation in DFU, but it could not explain the causal relationships between them. Thirdly, constrained by the retrospective study design, we did not explore the impacts of osteomyelitis, revascularization procedures, antibiotic therapy, glycemic treatment regimens, ulcer size and depth, peripheral perfusion status, handgrip strength and polypharmacy on amputation in DFU. Future prospective research is warranted to clarify the effects of these clinical factors. Fourthly, the follow-up period of 3 months was relatively short in the study, which may not be sufficient to capture delayed amputations or longer-term disease progression. Further prospective research with prolonged follow-up is needed to further evaluate the long-term prognostic applicability of the findings. Besides, our study focused on the Chinese population, and we need to enroll individuals from different countries and ethnic backgrounds to broaden the applicability of our conclusions in the future.

## 5 Conclusion

In conclusion, our findings indicated that inflammation and nutrition-derived indicators were independently associated with amputation in DFU. Among them, NHR and NAR had moderate predictive performance for the risk of amputation, which was not observed in previous studies. In addition, biomarkers including Wagner degree, LWR, HALP, monocyte, WBC, and NHR were selected to predict the risk of diabetic foot amputation using XGBoost model. It may help primary care physicians in early screening for individuals at high risk of amputation, which could guide targeted interventions to potentially lower amputation risk and improve the prognosis of patients with DFU. Since our study was only single-center study, multi-center external validation is necessary before it can be applied clinically.

## Data Availability

The original contributions presented in the study are included in the article/Supplementary material, further inquiries can be directed to the corresponding author/s.
