# Supplementary material for: Inflammation and nutrition-derived indicators for predicting amputation risk in patients with type 2 diabetic foot ulcers
Source: Front Nutr. 2026 Jun 30;13:1875176. doi: 10.3389/fnut.2026.1875176 (PMC13364899; doi:10.3389/fnut.2026.1875176)
Supplement: Supplementary file 1 [file Supplementary_file_1.DOCX]

Supplementary material

Supplementary Table S1 Baseline characteristics between included patients and excluded patients with type 2 diabetic foot ulcers.

| Variable | Included patients (n=1052) | Excluded patients (n=66) | P value |
| --- | --- | --- | --- |
| Age (years) | 64.01 ± 11.99 | 63.39 ± 11.2 | 0.684 |
| Gender (male), n (%) | 753 (71.6%) | 51 (77.3%) | 0.318 |
| Wagner degree, n (%) |  |  | 0.129 |
| I | 23 (2.2%) | 0 (0%) |  |
| II | 295 (28%) | 10 (15.2%) |  |
| III | 491 (46.7%) | 34 (51.5%) |  |
| IV | 241 (22.9%) | 22 (33.3%) |  |
| V | 1 (0.1%) | 0 (0%) |  |
| WBC (10^9/L) | 9.13 (7.15, 12.02) | 8.97 (7.08, 12.51) | 0.828 |
| Creatinine (mmol/L) | 93 (69, 132.75) | 89.1 (75, 136.8) | 0.76 |
| Uric acid（umol/L） | 332.85 (261.2, 421.75) | 338 (247.5, 395) | 0.7 |
| Albumin (g/L) | 33.7 (29.43, 37.1) | 32.3 (28.9, 36.2) | 0.124 |
| HbA1c (%) | 8.7 (7.1, 10.9) | 8.23 (7.1, 10.63) | 0.688 |

Abbreviations: WBC: white blood cells; HbA1c: glycated hemoglobin.

Supplementary Table S2 Univariate analysis between variables and amputation risk in diabetic foot ulcer.

| Variables | OR (95% CI) | P value | Tolerance | VIF |
| --- | --- | --- | --- | --- |
| Age | 0.984 (0.973,0.995) | 0.004 | 0.753 | 1.329 |
| Gender |  | 0.053 | 0.82 | 1.219 |
| Wagner degree | 8.056 (6.121, 10.602) | < 0.001 | 0.756 | 1.322 |
| smoking | 1.449 (1.052, 1.997) | 0.023 | 0.891 | 1.122 |
| CHD | 0.568 (0.378, 0.854) | 0.007 | 0.903 | 1.108 |
| PAD | 1.718 (1.312, 2.25) | < 0.001 | 0.801 | 1.248 |
| Hb | 0.981 (0.975, 0.987) | < 0.001 | 0.572 | 1.748 |
| Creatinine | 1.002 (1.001, 1.002) | < 0.001 | 0.794 | 1.259 |
| Uric acid | 0.998 (0.997, 0.999) | 0.003 | 0.803 | 1.245 |
| Albumin | 0.899 (0.878, 0.921) | < 0.001 | 0.534 | 1.873 |
| LDL-C | 0.71 (0.606, 0.832) | < 0.001 | 0.742 | 1.347 |
| HDL-C | 0.117 (0.069, 0.201) | < 0.001 | 0.678 | 1.475 |
| HbA1c | 1.093 (1.039, 1.15) | 0.001 | 0.838 | 1.193 |

Abbreviations: CHD: coronary heart disease; Hb: hemoglobin; LDL-C: low-density lipoprotein cholesterol; HDL-C: high-density lipoprotein cholesterol; HbA1c: glycated hemoglobin. VIF: Variance inflation factors.

Supplementary Table S3 Variance Inflation Factors (VIF) for covariates in separate logistic regression models for each inflammation and nutrition-derived indicator

| Indicators | VIF for indicator | VIF for Age | VIF for Gender | VIF for Wagner degree | VIF for smoking | VIF for CHD | VIF for PAD | VIF for Hb | VIF for creatinine | VIF for uric acid | VIF for albumin | VIF for LDL | VIF for HDL | VIF for HbA1c |
| --- | --- | --- | --- | --- | --- | --- | --- | --- | --- | --- | --- | --- | --- | --- |
| SIRI | 1.288 | 1.33 | 1.219 | 1.342 | 1.123 | 1.108 | 1.249 | 1.749 | 1.287 | 1.246 | 1.937 | 1.352 | 1.497 | 1.196 |
| NLR | 1.328 | 1.329 | 1.22 | 1.346 | 1.122 | 1.108 | 1.249 | 1.75 | 1.285 | 1.246 | 1.964 | 1.353 | 1.488 | 1.199 |
| PLR | 1.314 | 1.334 | 1.226 | 1.331 | 1.122 | 1.109 | 1.249 | 1.831 | 1.264 | 1.259 | 1.908 | 1.347 | 1.483 | 1.198 |
| PNR | 1.134 | 1.33 | 1.221 | 1.337 | 1.122 | 1.108 | 1.249 | 1.801 | 1.281 | 1.25 | 1.944 | 1.356 | 1.476 | 1.193 |
| NHR | 1.779 | 1.342 | 1.223 | 1.339 | 1.125 | 1.109 | 1.249 | 1.752 | 1.266 | 1.245 | 1.961 | 1.35 | 1.767 | 1.2 |
| MHR | 1.7 | 1.34 | 1.22 | 1.332 | 1.127 | 1.108 | 1.249 | 1.749 | 1.268 | 1.25 | 1.928 | 1.349 | 1.81 | 1.194 |
| PHR | 1.91 | 1.35 | 1.226 | 1.332 | 1.13 | 1.109 | 1.25 | 1.807 | 1.267 | 1.255 | 1.882 | 1.348 | 1.996 | 1.201 |
| LHR | 1.585 | 1.397 | 1.242 | 1.323 | 1.124 | 1.108 | 1.248 | 1.757 | 1.292 | 1.248 | 1.889 | 1.349 | 2.055 | 1.193 |
| NMR | 1.086 | 1.331 | 1.223 | 1.323 | 1.122 | 1.109 | 1.25 | 1.749 | 1.259 | 1.25 | 1.904 | 1.349 | 1.482 | 1.2 |
| LWR | 1.459 | 1.339 | 1.23 | 1.355 | 1.123 | 1.108 | 1.252 | 1.758 | 1.29 | 1.247 | 2.026 | 1.353 | 1.478 | 1.196 |
| NAR | 1.823 | 1.339 | 1.223 | 1.354 | 1.124 | 1.109 | 1.249 | 1.748 | 1.261 | 1.248 | 2.287 | 1.351 | 1.511 | 1.201 |
| HALP | 1.876 | 1.334 | 1.219 | 1.333 | 1.122 | 1.109 | 1.254 | 2.108 | 1.26 | 1.251 | 2.013 | 1.35 | 1.478 | 1.2 |

Abbreviations: VIF: Variance Inflation Factors; SIRI: systemic inflammation response index; NLR: neutrophil-lymphocyte ratio; PLR: platelet-lymphocyte ratio; PNR: platelet-neutrophil ratio; NHR: neutrophil-high-density lipoprotein ratio; MHR: monocyte-high-density lipoprotein ratio; PHR: platelet-high-density lipoprotein ratio; LHR: lymphocyte-high-density lipoprotein ratio; NMR: neutrophil-monocyte ratio; LWR: lymphocyte-white blood cell ratio; NAR: neutrophil-albumin ratio; HALP: the hemoglobin, albumin, lymphocyte, platelet score.

Supplementary Table S4 Sensitivity analysis for imputation-based data

| variables | Model 1 | | Model 2 | | Model 3 | |
| --- | --- | --- | --- | --- | --- | --- |
|  | OR (95% CI) | P value | OR (95% CI) | P value | OR (95% CI) | P value |
| SIRI | 1.099 (1.075,1.124) | < 0.001 | 1.099 (1.075,1.124) | < 0.001 | 1.045 (1.022,1.069) | < 0.001 |
| NLR | 1.126 (1.097,1.157) | < 0.001 | 1.126 (1.097,1.157) | < 0.001 | 1.058 (1.027,1.09) | < 0.001 |
| PLR | 1.004 (1.003,1.005) | < 0.001 | 1.004 (1.003,1.005) | < 0.001 | 1.002 (1.001,1.003) | 0.02 |
| PNR | 0.983 (0.977,0.989) | < 0.001 | 0.983 (0.977,0.989) | < 0.001 | 0.991 (0.985,0.998) | 0.009 |
| NHR | 1.083(1.065,1.102) | < 0.001 | 1.083(1.065,1.102) | < 0.001 | 1.044 (1.027,1.061) | < 0.001 |
| MHR | 2.88 (2.304,3.601) | < 0.001 | 2.88 (2.304,3.601) | < 0.001 | 1.818 (1.427,2.316) | < 0.001 |
| PHR | 1.003(1.002,1.003) | < 0.001 | 1.003(1.002,1.003) | < 0.001 | 1.001 (1.001,1.002) | 0.012 |
| LHR | 1.174 (1.041,1.324) | 0.001 | 1.139 (1.007, 1.288) | 0.039 |  | 0.594 |
| NMR | 1.051 (1.027,1.076) | < 0.001 | 1.049 (1.025, 1.074) | < 0.001 |  | 0.246 |
| LWR | < 0.001 (< 0.001, 0.001) | < 0.001 | < 0.001 (< 0.001, 0.001) | < 0.001 | 0.01 (0.001, 0.085) | < 0.001 |
| NAR | 44.013 (20.85, 92.907) | < 0.001 | 44.013 (20.85, 92.907) | < 0.001 | 7.363 (3.47,15.623) | < 0.001 |
| HALP | 0.949 (0.938,0.96) | < 0.001 | 0.949 (0.938,0.959) | <0.001 | 0.972 (0.96,0.984) | < 0.001 |

Notes: Logistic regression analysis of factors associated with amputation in DFU from imputation-based data. Model 1: Unadjusted; Model 2: Adjusted for age and gender; Model 3: Adjusted for age, gender, Wagner degree, smoking, CHD, PAD, Hb, creatinine, uric acid, albumin, LDL-C, HDL-C and HbA1c. P < 0.05 is considered statistically significant. Abbreviations: OR: odds ratio per original unit increase; SIRI: systemic inflammation response index; NLR: neutrophil-lymphocyte ratio; PLR: platelet-lymphocyte ratio; PNR: platelet-neutrophil ratio; NHR: neutrophil-high-density lipoprotein ratio; MHR: monocyte-high-density lipoprotein ratio; PHR: platelet-high-density lipoprotein ratio; LHR: lymphocyte-high-density lipoprotein ratio; NMR: neutrophil-monocyte ratio; LWR: lymphocyte-white blood cell ratio; NAR: neutrophil-albumin ratio; HALP: the hemoglobin, albumin, lymphocyte, platelet score.

Supplementary Table S5 The ROC test of different inflammation and nutrition-derived indicators for predicting amputation in DFU

| indicators | AUC (95% CI) | P value |
| --- | --- | --- |
| NAR | 0.756 | - |
| SIRI | 0.744 | 0.198 |
| NLR | 0.713 | < 0.001 |
| PLR | 0.678 | < 0.001 |
| NHR | 0.755 | 0.934 |
| MHR | 0.723 | 0.028 |
| PHR | 0.709 | 0.007 |

Notes: P value: Comparison of AUC value between NAR and other inflammation and nutrition-derived indicators by Delong’s test. Abbreviations: SIRI: systemic inflammation response index; NLR: neutrophil-lymphocyte ratio; PLR: platelet-lymphocyte ratio; NHR: neutrophil-high-density lipoprotein ratio; MHR: monocyte-high-density lipoprotein ratio; PHR: platelet-high-density lipoprotein ratio; NAR: neutrophil-albumin ratio.

Supplementary Table S6 Compare AUC differences among different machine learning models by Delong's test

| Model | AUC | P value |
| --- | --- | --- |
| XGBoost | 0.894 | - |
| Logistic regression | 0.892 | 0.688 |
| Random forest | 0.887 | 0.398 |
| SVM | 0.864 | 0.006 |
| LightGBM | 0.891 | 0.348 |

Abbreviations: SVM: Support Vector Machine; XGBoost: Extreme Gradient Boosting; LightGBM: Light Gradient Boosting Machine; AUC: area under the curve.
